# Long-term course of early onset developmental and epileptic encephalopathy associated with 2q24.3 microduplication

**DOI:** 10.1016/j.ebr.2022.100547

**Published:** 2022-04-25

**Authors:** Takuya Masuda, Hitoshi Osaka, Naomi Tsuchida, Satoko Miyatake, Kou Nishimura, Toshiki Takenouchi, Takao Takahashi, Naomichi Matsumoto, Takanori Yamagata

**Affiliations:** a Department of Pediatrics, Jichi Medical University, 2411-1 Yakushiji, Shimotsuke, Tochigi 329-0498, Japan; b Department of Human Genetics, Yokohama City University Graduate School of Medicine, 3-9 Fukuura, Kanazawa-ku, Yokohama 236-0004, Japan; c Department of Pediatric Neurology, Ise Keiyu Hospital, 2-7-28 Tokiwa, Ise, Mie 516-0041, Japan; d Department of Pediatrics, Keio University School of Medicine, 35 Shinanomachi, Shinjuku-ku, Tokyo 160-8582, Japan

**Keywords:** 2q24.3 microduplication, Developmental and epileptic encephalopathy, Voltage-gated sodium channel, Copy number variations, EIEEs, Early infantile epileptic encephalopathies, DEE, developmental and epileptic encephalopathy, SCN, voltage-gated sodium channels, PB, phenobarbital, VPA, valproic acid, ZNS, zonisamide, CZP, clonazepam, PHT, phenytoin, CLB, clobazam, WES, whole exome sequencing, CNVs, copy number variations

## Abstract

Copy number variations (CNVs) have been related to developmental and epileptic encephalopathy (DEE). The 2q24.3 region includes a cluster of genes for voltage-gated sodium channels (SCN) and CNVs in this region cause DEE. However, the long-term course of DEE with a 2q24.3 duplication has not been described. A 20-year-old female developed epileptic encephalopathy in early infancy that was resistant to various antiseizure medications. Her seizures disappeared after starting vitamin B6 therapy. Therefore, her epilepsy was considered pyridoxine-dependent epilepsy. At 16 years old, whole exome sequencing revealed a 2q24.3 microduplication including *SCN1A, SCN2A, SCN3A, SCN7A*, and *SCN9A*. Quantitative PCR detected an increased copy number of 1.3 Mb on 2q24.3 involving these genes, but no gene mutation accounting for pyridoxine-dependent epilepsy. Considering that with this duplication she was reported to be seizure-free after infancy, she was able to be off antiseizure medications including vitamin B6. Our case involvingdrug-resistant epilepsy in early infancy had no recurrent seizures during long-term follow up. Detecting CNVs using whole exome sequencing data was useful to identify a 2q24.3 duplication unassociated with pyridoxine-dependent epilepsy, leading to cessation of unnecessary medications.

## Highlights

• 2q24.3 is a region containing a cluster of genes for multiple voltage-gated sodium channels and CNVs in the region cause developmental and epileptic encephalopathy.
• DEE associated with 2q24.3 duplication have been reported to show early-onset epilepsy, resistant to antiseizure drugs, and severe developmental delay.
• Drug-resistant epilepsy due to 2q24.3 duplication can be seizure free after infancy.
• Our case was considered pyridoxine dependent but identifying duplication of 2q24.3 led to the discontinuation of unnecessary medication.

## 1 Introduction

Early infantile epileptic encephalopathies (EIEEs) are severe neurological disorders characterized by early-onset drug-resistant seizures, electroencephalographic abnormalities, and developmental delay [1], and are associated with structural brain defects, metabolic disorders, and genetic abnormalities [2]. In 2017, the ILAE classification of epilepsies suggested a new concept of developmental and epileptic encephalopathy (DEE), which are genetic epilepsies associated with developmental impairment as a direct consequence of a gene mutation [3], [4].

Causative genes of DEE have been reported by next-generation sequencing approaches [5], with more than 80 genes identified [6]. Among them, mutations in voltage-gated sodium channels (SCN), acting in cell depolarization and action potential propagation, have been implicated in DEE [7].

The chromosomal locus 2q24.3 is a region containing a cluster of genes for multiple SCN such as *SCN1A, SCN2A, SCN3A, SCN7A*, and *SCN9A.* The deletion of 2q24.3 has been reported to cause Dravet syndrome and focal seizures in infancy [8], while duplication of 2q24.3 has been reported to cause DEE, which is associated with early-onset epilepsy in the neonatal period, is resistant to antiseizure medication, and results in severe developmental delay [9]. The oldest patient reported previously was a 7-year-old with a mosaic duplication [10] and there are no long-term reports after infancy concerning 2q24.3 duplication-related DEE. Here, we report a 20-year-old female with DEE associated with a 2q24.3 duplication including *SCN1A, SCN2A, SCN3A, SCN7A*, and *SCN9A*.

## 2 Case report

The patient was a 20-year-old female, born at 39 weeks and 2 days of gestation without distress during the perinatal period. Her birth weight was 3075 g and her head circumference was 33.0 cm. She had no dysmorphic features and no particular family history of neurological disease.

On the first day after birth, she had seizures comprised of eye deviation and bicycling movements. She showed bradycardia and cyanosis during her seizures. Blood chemistry, blood gas analysis, brain CT, and MRI showed no abnormalities. EEG showed spike-and-waves in the right parietal lobe and diffuse spike-and-wave bursts in the right cerebral hemisphere. A combination of antiseizure medications with phenobarbital (PB), valproic acid (VPA), zonisamide (ZNS), and clonazepam (CZP) were effective, and she was discharged at 8 weeks old.

At 14 weeks old, she had generalized tonic-clonic seizures with apnea and eye deviation lasting 1–2 min and was hospitalized. As seizures occurred frequently (10 times/day), she was intubated under sedation. Although phenytoin (PHT) was started and CZP was changed to clobazam (CLB), seizures were not controlled. Brain MRI at 5 months old showed atrophy of the frontal lobe and enlargement of the sulci. Interictal EEG revealed focal spikes and sharp waves on the right central to parietal regions, and spikes focused on the left central and slow wave on the left frontal regions. Considering her interictal EEG findings and course, we diagnosed her with DEE with focal to bilateral tonic-clonic seizures. Since her epilepsy was drug-resistant, PHT was discontinued and oral pyridoxin of 30 mg/kg/day was started. Soon after pyridoxin was started, her seizures disappeared. She was discharged at 6 months old and had no further seizures. Her developmental milestones were delayed (sit alone at 8 months, roll over at 1 year 2 months, and walk with support at 3 years of age). She had intermittent exotropia, increased deep tendon reflex, and her muscle tones were normal.

**Fig. 1:**
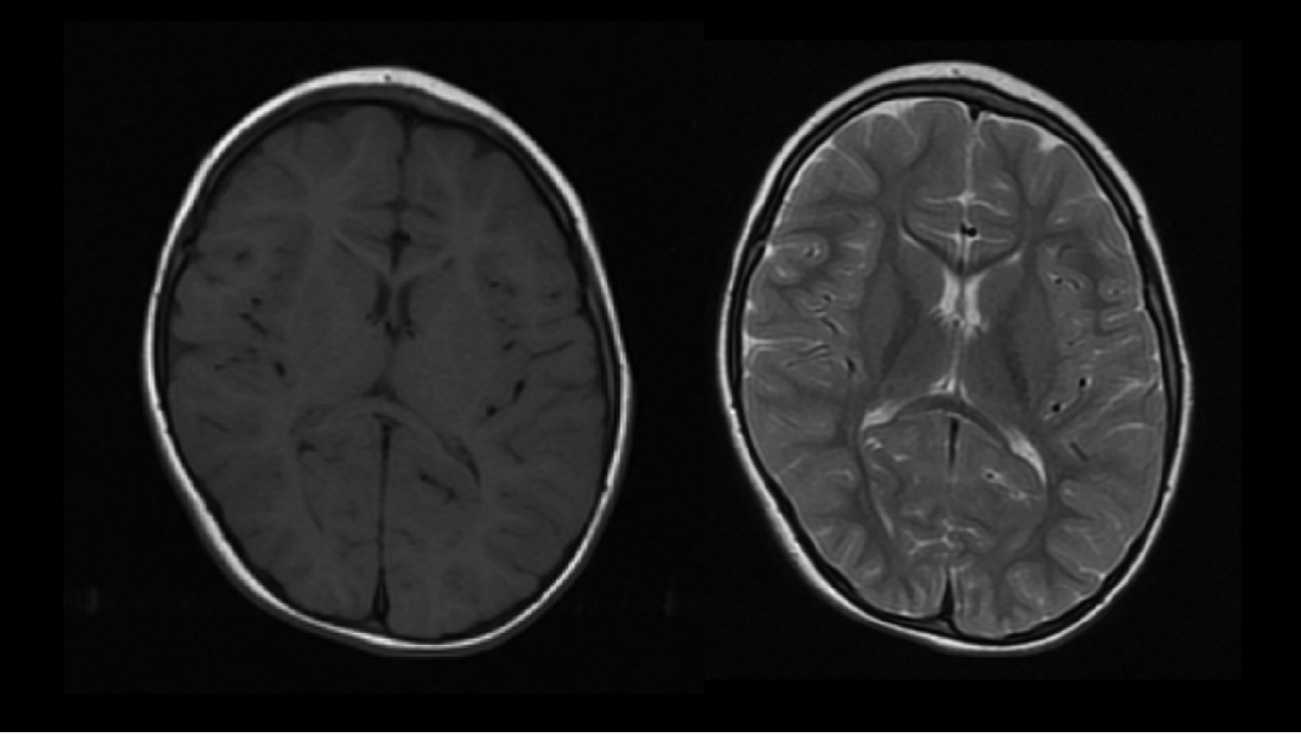
MRI Axial T1/T2 MRI images at 4 years old showed mild atrophy of the frontal cortex and thinning of the corpus callosum. Myelination was complete. No structural abnormalities such as cortical dysplasia were observed.

At the age of 1 year and 5 months, she discontinued ZNS. Brain MRI at 4 years old showed mild atrophy of the frontal cortex and thinning of the corpus callosum, but no cerebellar atrophy (Fig. 1). EEG at 6 years old showed prominent beta activity and normal background activity, without epileptic discharges. Although her seizures did not recur, she continued to take antiseizure medications and pyridoxin.

When she was referred to our hospital at the age of 7 years and 6 months, she was in the second grade in a special needs school. She showed ataxic gait, equinovarus foot, and ankle clonus. She was able to express her intentions in simple words and to feed herself independently. Since her seizures disappeared completely at the time of pyridoxin initiation, we considered her epilepsy vitamin B6 dependent. At 14 years old, we started a reduction of VPA to 100 mg/day (4 mg/kg/day), but did not further reduce antiseizure medications because her mother worried about recurrence of her seizures.

**Fig. 2:**
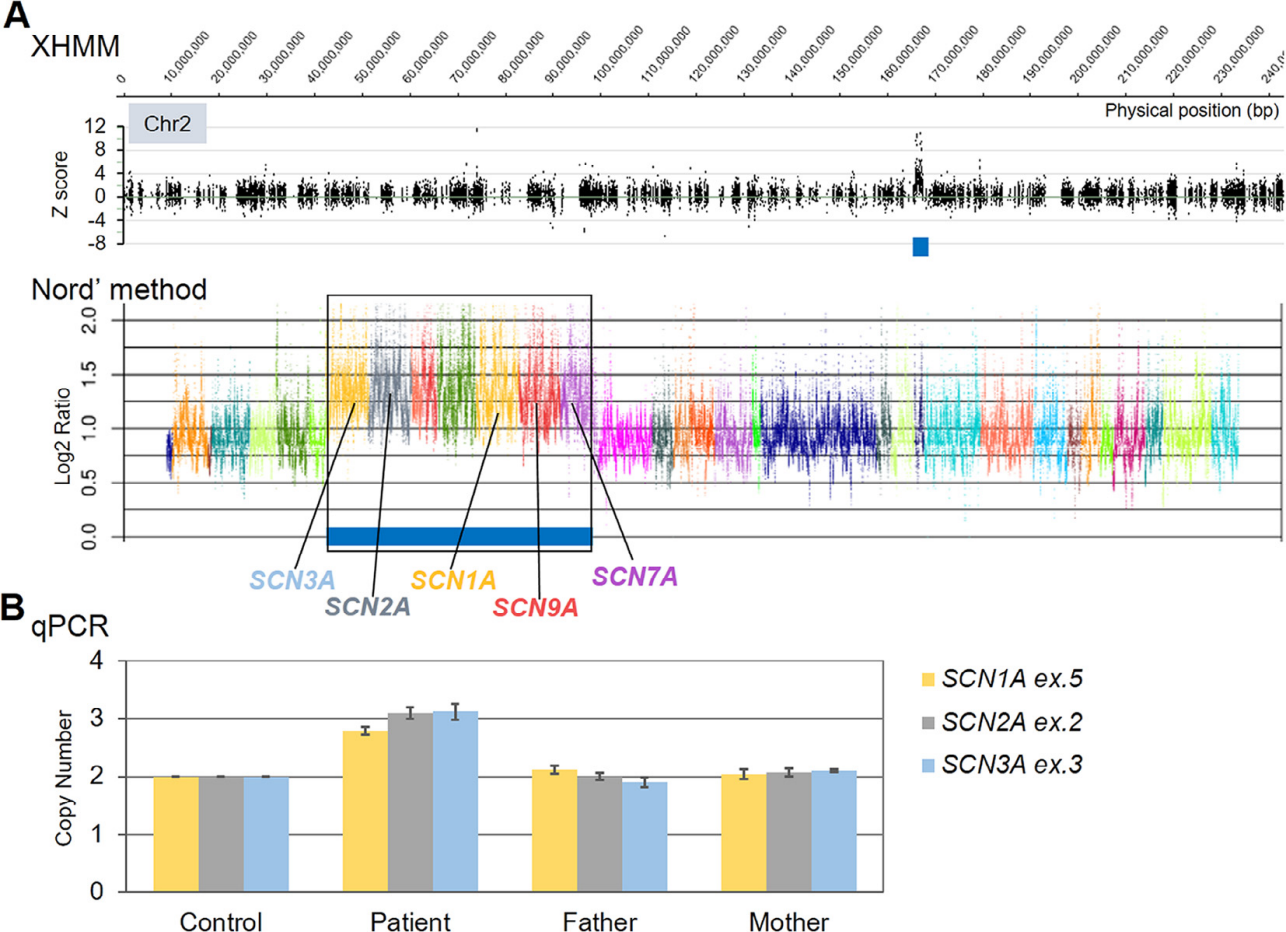
A de novo 1.3-Mb duplication at 2q24.3 in the patient detected by WES-based programs. (A) Results of CNV analyses by XHMM and Nord’s method. For the XHMM, the X axis shows the genomic position and the Y axis indicates the Z score for each target. For the Nord’s method data, the X-axis shows arrays of targeted genes with different colors, with their proportional physical length, and the Y-axis shows log2 ratios for each targeted base in the genes tested. The blue thick bars represent calls for copy number gains in each method. (B) Results of qPCR analysis. The X-axis shows respective samples and the Y-axis shows the relative copy number. Target genes for qPCR are shown to the right of the bar graphs. A normal control and family members were used for the qPCR. CNV, copy number variation; Nord’s method, program based on relative depth of coverage ratios developed by Nord et al. [19]; qPCR, quantitative polymerase chain reaction; WES, whole-exome sequencing; XHMM, eXome Hidden Markov Model. (For interpretation of the references to colour in this figure legend, the reader is referred to the web version of this article.)

After obtaining informed consent, whole exome sequencing (WES) was performed at 16 years of age as previously described [11]. Including *ALDH7A1*, there were no pathogenic gene mutations accounting for her epileptic encephalopathy (*i.e., SCN8A*)*.* Instead, a 2q24.3 microduplication that included *SCN1A, SCN2A, SCN3A, SCN7A,* and *SCN9A* was detected in the patient (Fig. 2A). Quantitative PCR revealed an increased copy number of 1.3 Mb at 2q24.3 (Fig. 2B). While healthy controls and her parents had 2 copies, the patient had 3 copies (*de novo*). We stopped vitamin B6, as well as CLB and VPA at 17 years. At present, she is 20 years old and free of seizures without antiseizure medications. She is able to walk alone and communicate with others using her own simple words, but her IQ is less than 35.

## 3 Discussion

We report the case of a 20-year-old female with DEE associated with a 2q24.3 microduplication. During early infancy, her epilepsy was resistant to six different antiseizure medications. However, she has been seizure-free since 6 months of age. Our case never showed recurrence of seizures, was able to walk by herself, and could communicate with simple words. Our long-term follow up case reinforces the notion that patients with a 2q24.3 duplication can be free of seizures and antiseizure medications after early infancy [9].

Our case carries duplication of *SCN1A, SCN2A, SCN3A, SCN7A,* and *SCN9A* in 2q24.3. Previous reports suggest that duplications of *SCN2A* and *SCN3A* play a crucial role in the phenotype of patients with 2q24.3 duplication: neonatal-onset seizure, relatively better seizure outcome, and delayed development (Supplementary Figure). Patients with 2q24.3 duplication have been reported to show normal MRI, but there is a report of a case of frontal lobe atrophy [12]. In that report, one case showed moderate atrophy of bilateral frontal lobes at the age of 18 months. Her development was severely delayed, with no head control at the time. Frontal lobe atrophy may be related to the severe intellectual disability.

Since her seizure disappearance coincided with the start of vitamin B6 therapy, her epilepsy was regarded as pyridoxine responsive at that time. Pyridoxine-dependent epilepsy is an epileptic encephalopathy caused by antiquitin deficiency due to mutations in *ALDH7A1* [13]. This epilepsy shares similarity with 2q24.3 duplication in neonatal seizure onset and resistance to antiseizure medications [14]. In previous reports, 3 patients with 2q24.3 duplication were treated with vitamin B6 without effect [9], [12]. In the present case, seizures were not vitamin B6 dependent as she did not show any seizures after the vitamin B6 withdrawal [15]. It is possible that the time of her seizure disappearance from a natural course of 2q24.3 duplication coincided with the timing of the start of vitamin B6. However, vitamin B6 might contribute to seizure remission, as some drug-resistant epilepsies have been reported to respond to pyridoxine [16]. Considering her drug-resistant seizures during early infancy and the timing of seizure control, we continued her antiseizure medication until 16 years of age. WES showed that she carried copy number variations (CNVs) with no variations for *ALDH7A1*, which allowed the termination of her medications. The high resolution chromosomal microarray approach has discovered novel rare DNA CNVs across the genome and identified many causative genes. WES has recently been applied to detecting CNVs by both XHMM and Nord’s method using WES data [17], [18]. This approach appears useful to increase diagnostic yield to identify genetic causes of epilepsy, as in this case.

## 4 Conclusion

We report the long-term course of DEE associated with a 2q24.3 duplication. After the diagnosis at 16 years old, she was able to be free of antiseizure medication including vitamin B6. Elucidation of a 2q24.3 duplication and epilepsy appears beneficial as it may enable the cession of unnecessary medications.

## Ethical statement

Written informed consent was obtained from the parents for performing next generation sequencing and publication of this case report. This study was approved by the ethical committee of Jichi Medical University (G20-ver12).

## CRediT authorship contribution statement

**Takuya Masuda:** Writing – original draft. **Hitoshi Osaka:** Conceptualization, Writing – review & editing, Supervision. **Naomi Tsuchida:** Visualization, Investigation. **Satoko Miyatake:** Investigation. **Kou Nishimura:** Investigation. **Toshiki Takenouchi:** Investigation. **Takao Takahashi:** Investigation. **Naomichi Matsumoto:** Supervision, Investigation. **Takanori Yamagata:** Writing – review & editing, Supervision.

## Declaration of Competing Interest

The authors declare that they have no known competing financial interests or personal relationships that could have appeared to influence the work reported in this paper.
