# Supplementary material for: Long-term course of early onset developmental and epileptic encephalopathy associated with 2q24.3 microduplication
Source: Epilepsy Behav Rep. 2022 Apr 25;19:100547. doi: 10.1016/j.ebr.2022.100547 (PMC9207545; doi:10.1016/j.ebr.2022.100547)
Supplement: Supplementary data 1 [file mmc1.pptx]

## Slide 1
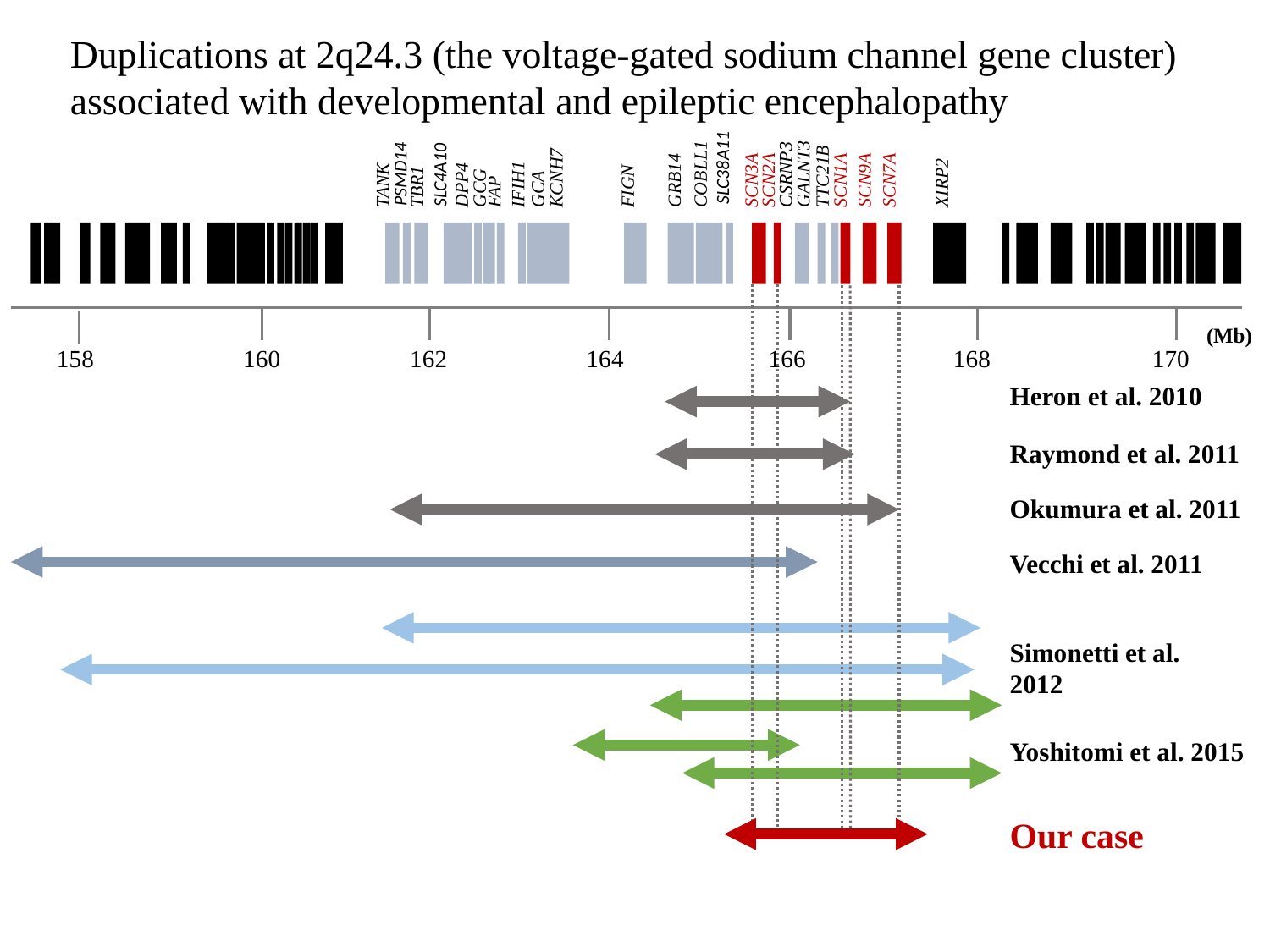

Duplications at 2q24.3 (the voltage-gated sodium channel gene cluster)
associated with developmental and epileptic encephalopathy
SLC38A11
PSMD14
SLC4A10
TANK
TBR1
DPP4
GCG
FAP
IFIH1
GCA
KCNH7
FIGN
GRB14
COBLL1
SCN3A
SCN2A
CSRNP3
GALNT3
TTC21B
SCN1A
SCN9A
SCN7A
XIRP2
(Mb)
158
160
162
164
166
168
170
Heron et al. 2010
Raymond et al. 2011
Okumura et al. 2011
Vecchi et al. 2011
Simonetti et al. 2012
Yoshitomi et al. 2015
Our case
